# Examining Sex-Based Delays in Utilizing Advocacy Support Services Among Australian Military Veterans: Implications for Health Care Access and Suicide Prevention

**DOI:** 10.3390/ijerph21111467

**Published:** 2024-11-03

**Authors:** Andrew Prevett, Monica Short, Maxwell Morrissey, Ben Wadham

**Affiliations:** 1 College of Education, Psychology and Social Work, Flinders University, Adelaide, SA 5042, Australia; ben.wadham@flinders.edu.au; 2 Open Door Initiative: Improving the Wellbeing of Veterans and Public Safety Personnel and Their Families, Flinders University, Adelaide, SA 5042, Australia; 3 School of Social Work and Arts, Charles Sturt University, Wagga Wagga, NSW 2678, Australia; mshort@csu.edu.au (M.S.);

**Keywords:** military veteran, advocacy, help-seeking behavior, suicide, stigma, military culture

## Abstract

This study explores the impact of sex on delays experienced by Australian military veterans in accessing advocacy support services within the veterans’ non-profit sector. A detailed analysis of intake records from 150 injured veterans who entered the Returned and Services League of Australia’s advocacy program in 2021 reveals significant disparities between male and female veterans in seeking assistance. On average, male veterans delay accessing support by 20.4 years post-service, compared to 9.1 years for female veterans. These prolonged delays hinder veterans’ ability to secure financial and medical support from the Department of Veterans’ Affairs, limiting timely access to essential healthcare services. As a result, delays can exacerbate physical and psychological symptoms, impede recovery, and increase the risk of suicide. The findings provide valuable insights for international healthcare professionals on the influence of military culture and traditional masculine norms in shaping veterans’ help-seeking behaviors. By understanding these dynamics, healthcare practitioners can develop targeted, sex-sensitive interventions that address specific barriers faced by male and female veterans. Ensuring timely access to advocacy support is crucial for improving health outcomes and reducing suicide risk in this vulnerable population.

## 1. Introduction

When military personnel separate from the Australian Defence Force (ADF), they encounter the complex task of navigating various domains to ensure a successful transition and reintegration into civilian life. One particularly crucial area is the Department of Veterans Affairs (DVA) injury claims process, which often involves seeking guidance from an advocate. However, many veterans decide to delay their participation in this process, often returning years later when their health and well-being has declined.

As per the 2021 Census of Population and Housing by the Australian Bureau of Statistics, there are around 496,000 living ex-serving military veterans in Australia, each with diverse demographic characteristics and health statuses [1,2]. In 2023, it was reported that 240,000 of these veterans received financial assistance or medical treatment funded by DVA, indicating that 48.3% had liability accepted by DVA for at least one service-related injury [3]. Since 2019, DVA has observed an annual increase in the number of claims received, with a growth rate of about 13% per year [4]. This rise can be attributable to the significant number of ADF warlike operations and deployments since 1999, as well as the introduction of DVA’s new online claiming platform, ‘MyService’, in 2017 [5]. This surge in claims has led to delays in determining veterans’ eligibility for crucial healthcare funding, counselling, and other supports, increasing the risk of suicide and psychological distress among some veterans [6]. Of particular concern to healthcare workers internationally is that early access to healthcare, including mental health care, can significantly reduce the risk of suicidality among veterans. Conversely, delayed access can exacerbate existing health conditions and increase associated mortality rates [5,7,8].

Although research on veterans’ health and well-being exists, limited international attention has been given to the delaying behavior of veterans before submitting an injury recognition claim and the potential influence of sex on these patterns. Additionally, there is a noticeable lack of research focusing on the health and well-being of female veterans in the Australian context, despite women comprising 20.6% of the ADF workforce [9,10]. This study explores the following research question: how does identifying as male or female affect the delay in veterans seeking advocacy support? By examining the intake data of veterans entering the South Australian Returned and Services League (RSL-SA) advocacy support program, the study aims to enhance the understanding of sex differences and uncover significant patterns and disparities.

The findings from this research can guide healthcare workers in delivering customized preventative health education and therapeutic support to veterans injured through their service, especially those lacking access to necessary healthcare funding and support from DVA. Furthermore, the insights gained can aid practitioners in creating sex-specific intervention programs, thereby more effectively addressing the needs of this vulnerable veteran population.

## 2. Engaging the Literature

### 2.1. Veteran Suicide Statistics

From 1985 to 2021, 83% of the 2007 recorded suicides among individuals with ADF service were ex-serving veterans [11,12]. On average, at least three serving or ex-serving veterans die by suicide every fortnight [11]. After adjusting for age, the suicide rates for male veterans were 27% higher, and for female veterans, 107% higher compared to their counterparts in the Australian general population [13]. These trends align with broader international patterns observed among the Five Eyes nations. In 2021, U.S. male veterans had a suicide rate 43.4% higher than the general population, while female veterans were 166.1% higher [14]. In Canada for 2021, male veterans had a 50% higher suicide rate, and female veterans had a 100% higher rate compared to their civilian counterparts [15]. New Zealand’s veteran suicide rates were similar to those of Australia’s, with ex-serving male veterans experiencing a 21% higher rate and female veterans facing a 127% higher rate between 2019 and 2021 [16]. The UK is an outlier; due to the predominantly male veteran population, analysis mainly focuses on male suicides, revealing no overall difference in suicide rates between male UK armed forces veterans and the general male population [17]. However, in 2021, the suicide rate for male UK veterans aged from 25 to 34 was 112% higher, and for those aged from 35 to 44, it was 78% higher [17]. These figures demonstrate a persistent pattern of elevated suicide rates among military veterans across various countries.

Among Australian veterans who died by suicide, half did so 20 years or more after their discharge from the ADF, and just under a quarter had served between 10 and 20 years before their suicide [18]. Of the ex-serving veterans who died by suicide from 2002 to 2021, 70.9% of males and 75.2% of females were not DVA clients at the time of their death [12]. This data indicates that most veterans who die by suicide are males, discharged from the ADF on average 20 years prior, and were not receiving essential healthcare funding and other supports provided by DVA [18]. In this context, advocacy services that facilitate connections between veterans and DVA resources are important for mitigating suicide risk. Promoting timely engagement with advocacy services could ensure that veterans receive the necessary support they need to improve their overall health and well-being, thereby potentially reducing the likelihood of suicide.

### 2.2. Help-Seeking Behavior, Essential Healthcare, and Suicide Risk

Understanding the risk factors associated with suicide is inherently complex, particularly among veterans, as these factors are shaped by a combination of individual, social, and contextual elements [19]. Despite this complexity, research consistently highlights a significant correlation between untreated mental health disorders and an elevated risk of suicide in this population [19,20,21]. For instance, a report by the Australian Institute of Health and Welfare indicates that from 2001 to 2018, 83% of veterans who died by suicide had a mental health or behavioral condition, with 77% not seeking support from healthcare services in the year prior to their death [22]. This lack of help-seeking behavior is further highlighted by a systematic review indicating that between 40% and 60% of veterans who could benefit from formal mental health treatment were not accessing the necessary care [23].

Delays in seeking help can exacerbate existing conditions, leading to poorer health outcomes [24,25]. The Royal Commission into Defence and Veteran Suicide (2023) identified that such delays contribute significantly to veterans’ stress, mental ill-health, and increased risk of suicidality [5]. Furthermore, data reveal that two-thirds of ex-serving veterans who died by suicide between 2002 and 2021 were not registered as clients with DVA, suggesting that a substantial number of at-risk veterans are not filing injury recognition claims to the department [12,22].

Submitting injury claims to DVA after significant time impedes timely access to essential healthcare services and resources, potentially exacerbating both physical and mental health conditions, extending recovery periods, and elevating the risk of suicide among veterans. When access to timely healthcare is delayed, veterans’ overall well-being can deteriorate, leading to a harmful cycle of deteriorating health [24,25]. The absence of prompt care and support often leads to feelings of isolation and helplessness, contributing to heightened despair and increasing the likelihood of suicidal thoughts or actions [26].

### 2.3. Advocacy Support Services

Advocacy and welfare support services for Australian veterans are primarily provided by advocates accredited through the Advocacy Training and Development Program (ATDP), operating within the veterans’ non-profit sector [27]. In 2019, there were 538 advocates nationally, with 417 trained in compensation and the remainder in well-being [27]. Each type of advocate plays a unique role: compensation advocates assist veterans in preparing DVA injury claims, obtaining medical evidence, representing them at appeals, and acting as intermediaries between DVA staff and veterans, while well-being advocates offer support for veterans experiencing personal, family, or health problems, such as addiction, employment, accommodation, and finance [28].

In Australia, the Returned and Services League (RSL) employs the majority of advocates in this field and is the largest ESO in terms of membership and geographical size, with an extensive network of 1135 sub-branches across Australia [29]. The RSL-SA is coordinated from its Adelaide office and manages approximately 90 sub-branches across South Australia, the Northern Territory, and Broken Hill in New South Wales [30]. Due to the complexity of the claims process, many veterans seek the support of a sector advocate, such as those within the RSL network.

Access to timely advocacy is essential in reducing suicide risk among veterans, especially as risk factors for suicidality tend to accumulate and intensify over time [24,25]. The final report of the Royal Commission into Defence and Veteran Suicide (2024) emphasized that many veterans who died by suicide were not receiving support from DVA at the time [31]. This highlights the urgent need for effective advocacy services that connect veterans to essential healthcare. By facilitating timely access to healthcare, mental health services, and financial assistance, advocates can help alleviate factors that contribute to suicidality [32].

Early intervention through advocacy is especially important during the challenging transition from military to civilian life [33]. Advocates play a key role in encouraging veterans to seek the support they need. Ensuring that veterans can successfully navigate the claims process and access assistance is vital for promoting good mental health and well-being. This support not only fosters a culture of help-seeking but also expedites access to critical resources, mitigating risk factors associated with suicide.

## 3. Methodology

### 3.1. Definition of Sex

To ensure clarity, it is essential to explicitly define the term ‘sex’ as used in this research. Sex refers to a set of biological characteristics associated with physical and physiological traits, such as chromosomal genotype, hormonal levels, and internal and external anatomy. This research employs a binary categorization of sex (male/female), which aligns with the designation typically assigned at birth.

### 3.2. Data Source

The intake questionnaires of veterans seeking support from an RSL-SA compensation advocate underwent detailed quantitative analysis. These questionnaires were designed and implemented by RSL-SA to collect intake information from veterans accessing their advocacy services. The authors were not involved in the creation or the distribution of the questionnaire and did not receive copies from RSL-SA. A de-identified dataset containing intake information from these questionnaires in 2021 (*n* = 150) was provided to the researchers for use in this study. This dataset included information on the veterans’ ADF service history, injury classification, self-reported usage rates of previous advocacy support services, and socio-demographic characteristics. Data were collected in a confidential electronic format, with identifying details redacted to preserve anonymity. The secondary analysis of this data was approved by both the Charles Sturt University Human Research Ethics Committee (H22201) and the RSL-SA, adhering to the guidelines set forth in the National Statement on Ethical Conduct in Human Research.

### 3.3. Sample Selection

The study was limited to veterans who met the inclusion criteria: completed the RSL-SA initial intake questionnaire in 2021 and being assigned to an advocate for the purpose of submitting an initial liability injury claim to DVA. The sample comprised of 150 veterans, including 108 males and 42 females.

## 4. Measures

### 4.1. Independent Variable

The independent variable of interest in this study is sex, dichotomized within the data source as either male or female.

### 4.2. Dependent Variable

The dependent variable is the length of delay between discharge and the seeking of advocacy support services.

### 4.3. Potential Confounders

Confounders available for the entire study sample included: length of service, service branch, highest achieved rank, discharge type, age on advocacy engagement, injury category, and deployment status.

### 4.4. Data Analysis

The study employed simple descriptive analysis to calculate counts and proportions for categorical data, as well as mean and standard deviation for continuous data. The independent variable of interest was sex, categorized as male or female for this study. A sex-stratified univariable analysis was conducted to assess differences in the length of delays associated with potential confounders. Normality of the dependent variable data was confirmed using the Kolmogorov-Smirnov (K-S) test. Given variations in sample sizes and variances between male and female groups, continuous variables were analyzed using Welch’s *t*-test to determine statistical significance (*p* < 0.05). All analyses were conducted using SPSS software, version 9.4. The length of delay before seeking advocacy support was calculated by subtracting the year of ADF discharge from the year of advocacy support service utilization, which for this sample was uniformly set at 2021 due to no previous reported engagements with advocacy services.

The length of service was placed into 5 categories: 1–5, 6–10, 11–15, 16–20 and 21+. These categories were chosen to show the diversity within this confounder. Age distributions in the sample were categorized as 18–32 and 33+ years to distinguish between contemporary and older veterans. For consistency reflecting the predominant Army service in the sample, equivalent ranks from all service branches were grouped using Army ranks. Ranks with low numbers (<5) were merged with comparable levels, such as Lance Corporal with Corporal and Staff Sergeant with Sergeant. The two commissioned officers in the sample were categorized together.

## 5. Results

Table 1 presents the results of the descriptive statistics for the variables and potential confounders used in the analysis. A total of 150 veterans (72% male/28% female) were included in the study. The results show that the majority of the sampled veterans had served in the Army (60%), achieved a junior rank of Corporal or below (88%), were older than 33 years when initially engaging with an advocacy service (94%), and had not deployed (83%). Although the majority of the sample had a length of service below 10 years (83%), male veterans had a higher average service length of $\bar{x}$ 8.1 ± 6.7 compared to female veterans of $\bar{x}$ 5.5 ± 3.4. In addition, male veterans had greater levels of voluntary discharge from the ADF (86%) while females had a close to even distribution between the two reported discharge types (52%/47%). Male veterans were also more likely to self-report a mix of psychological and physical injuries (62%) while female veterans self-reported physical only injuries at higher rates (59%). Neither sex reported the presence of psychological only injuries.

### Length of Delay in Accessing Support

According to Table 2, sex was a significant predictor of delaying behavior in seeking advocacy support among the sampled Australian veterans, with male veterans experiencing longer delays than female veterans across all analyzed confounders. With about 80% of male veterans having a length of delay greater than 16 years while 73.8% of female veterans having a delay under 10 years. Analysis of the confounders of length of service and highest rank achieved also revealed significant differences (*p* < 0.001) in delay length between male and female veterans. For example, male veterans with less than 15 years of service experienced delays of $\bar{x}$ 20.4 years, compared to delays of $\bar{x}$ 10 years for female veterans. Additionally, male veterans that achieved a junior rank of Corporal or below experienced a delay of $\bar{x}$ 20.1 years, compared to a delay of $\bar{x}$ 8.9 years for female veterans. However, the level of significance was slightly reduced (*p* < 0.002) for veterans at the rank of Sergeant, with male veterans experiencing a delay of $\bar{x}$ 23.3 ± 3.2 and female veterans experiencing delays of $\bar{x}$ 13.0 ± 1.4. A general trend was observed for delay length to increase alongside length of service and rank achievement for both sexes.

This pattern of significance (*p* < 0.001) was also observed for service branch (Army and Air Force), injury category, deployment status, discharge type, and age above 33 at time of engaging with advocacy services. The level of significance was slightly reduced (*p* 0.005) for veterans who had deployed, with male veterans experiencing delays of $\bar{x}$ 20.4 ± 3.7 and female veterans experiencing delays of $\bar{x}$ 9.0 ± 3.6. In analyzing the Navy service branch and veterans aged between 18–32 at the time of advocacy support engagement, the length of delay was not statistically significant at *p* = 0.123 and *p* = 0.299, respectively. However, as per Table 1, these results may have been influenced by the small sample sizes in these groups.

## 6. Discussion

This study highlights that the military institution, with its unique culture and values, significantly influences the behaviors and decisions of its members [34]. It corroborates existing literature indicating that military culture places a strong emphasis on institutional loyalty over individual safety, which can foster risk-taking behaviors increasing the likelihood of injuries and other adverse outcomes [35,36]. While promoting loyalty and teamwork, this culture may discourage injured members from reporting injuries or seeking healthcare due to fear of stigma and potential career repercussions [37,38]. Negative stereotypes, prejudice, and discrimination against injured members further exacerbate these concerns, creating barriers to seeking necessary support [32,37].

Military training and socialization reinforce these beliefs and behaviors, shaping the identities and lifestyles of its members both during and after their service [39]. Veterans often carry these military identities into civilian life, impacting their willingness to seek advocacy support and preventative healthcare services, particularly for males who have longer exposure to military culture [39].

Research on sex differences in help-seeking behavior and healthcare utilization among veterans is limited, especially in the Australian context. Existing literature suggests that, compared to males, females generally hold more positive attitudes towards professional help-seeking and are more inclined to recognize their personal need for assistance [40]. Femininity appears to play a significant role in shaping these attitudes, influencing how individuals perceive and tolerate stigma associated with seeking help [41]. Studies indicate that females are more accepting of help-seeking, more likely to acknowledge their need for assistance, and more open about sharing their challenges [42]. Female veterans have been found to be 1.5 times more likely than male veterans to engage actively in their health and healthcare [43]. Conversely, adherence to traditional masculine norms can hinder male willingness to seek help, particularly for injuries [44]. Males who conform closely to these norms, including those within the military, may view help-seeking as a sign of weakness [45,46], what Wadham et al. [33] labeled as vulnerability stigma. The pervasive societal stigma around mental health also impacts both sexes within the veteran community, potentially hindering help-seeking behavior. Additional factors include potential feelings of bitterness or resentment toward their service that some veterans may harbor and take time to resolve.

This study’s findings reveal that male veterans in the sample experience significantly longer delays in seeking advocacy support compared to female veterans. Factors contributing to this difference likely include the influence of military culture and masculine norms on male veterans, alongside the more proactive help-seeking behaviors and greater tolerance of stigma observed among female veterans. Further research is necessary to fully understand these factors and develop targeted interventions to address disparities in help-seeking among veterans.

## 7. Limitations

This study is subject to several limitations that must be considered. First, the sample was limited to a convenience sample of veterans seeking assistance from an advocate in Adelaide, South Australia. Additionally, all veterans in the study sought assistance from the RSL-SA advocacy support service, which is not the only provider of advocacy services to veterans. Furthermore, as the data were collected from intake questionnaires, there is no information on whether or not an injury claim was actually submitted or the outcome of any submissions. Another point to consider is that the data span only 12 months, restricting the study’s generalizability and the ability to assess long-term trends.

Another limitation is that the data were collected from a questionnaire developed and implemented by a third party (RSL-SA) rather than an expertly validated instrument, potentially affecting the reliability of the data. Finally, although the proportion of females in this research closely mirrors their representation within the ADF, their low proportion in the study’s sample adds to the error in inference, making it difficult to draw firm conclusions regarding sex-based differences. Despite the study’s limitations, it offers valuable insights into the challenges that veterans encounter when seeking support and sheds light on sex differences in delaying behavior.

## 8. Conclusions and Implications for Health Care

An important implication for healthcare practice and research is recognizing the impact of sex on veterans’ propensity to delay seeking advocacy support services and the subsequent implications for accessing appropriate healthcare funding, particularly in light of the elevated risk of suicide among veterans. The study’s finding that male veterans tend to delay seeking support longer than their female counterparts underscores the necessity for targeted, sex-sensitive interventions that address the distinct challenges male veterans face in accessing adequate care. However, while female veterans exhibit shorter delays compared to males, their overall delay in seeking help from an advocate remains unacceptably high. This indicates that female veterans also encounter similar influential factors as their male counterparts. Therefore, healthcare practitioners can play a crucial role in identifying and addressing these challenges through sex-sensitive approaches that acknowledge potential barriers to help-seeking, such as traditional gender roles, stigma, and military culture.

To strengthen future research, increasing the sample size and ensuring better representation of female veterans will be essential for drawing more reliable inferences. Additionally, incorporating qualitative interviews can provide valuable insights by confirming conceptual links between traditional gender roles, stigma, and military culture. While the results of this study indicate similar cultural values across military branches, some variations exist, suggesting potential differences in values and cultural norms that could influence help-seeking behaviors and, consequently, health outcomes. Therefore, future research should systematically investigate the extent and significance of these variations across different service branches and perform cross-national comparisons. This approach will enhance understanding of how these factors influence veterans’ health-seeking behaviors and health outcomes in various contexts.

It is also essential to examine the paradox faced by female veterans, who experience higher rates of suicide and lower engagement with essential DVA services prior to their deaths compared to their male counterparts, despite seeking advocacy support more rapidly. Investigating this phenomenon may involve exploring the impact of social support networks and the urgency of crisis situations on their help-seeking behaviors. Understanding these dynamics can yield valuable insights into effectively addressing the unique needs of female veterans.

By recognizing the influence of sex on veterans’ help-seeking delays and its impact on their access to appropriate healthcare funding is crucial for advancing both healthcare practice and research. By acknowledging and addressing the distinct needs and experiences of veterans, healthcare practitioners, researchers, and policymakers can collaborate to develop interventions that facilitate timely and effective access to care, thereby promoting long-term well-being and mitigating the risk of suicide among veterans.

## 9. Recommendations

- Develop targeted educational programs to reshape male veterans’ perceptions of vulnerability and help-seeking, emphasizing these traits as strengths. These programs should leverage peer mentors with shared military backgrounds to encourage timely engagement with healthcare and advocacy services.
- Design outreach initiatives for female veterans that address their unique service experiences, fostering supportive networks that empower them to confidently seek healthcare and advocacy assistance.
- Implement sex-specific advocacy programs that confront stigma and cultural barriers preventing veterans from seeking help, tailored to address the distinct physical and mental health needs of both sexes.
- Integrate DVA services and resources into transition programs, while expanding outreach through workshops and information sessions to ensure all veterans, both male and female, are fully informed about available healthcare, advocacy support, and funding pathways.
- Expand military cultural competency training for healthcare providers, with DVA-funded incentives, to improve understanding of veterans’ unique healthcare challenges, including sex-specific and service-related issues.
- Strengthen partnerships with healthcare organizations to promote the widespread adoption of military cultural competency, ensuring that veterans’ service backgrounds and sex-specific challenges are recognized and addressed in healthcare services.

## Figures and Tables

**Table 1:** Descriptive statistics of variables used in the analysis, stratified by sex (*n* = 150).

Variables/Confounders	Male		Female	
	*n*	Mean (SD)/Percent	*n*	Mean (SD)/Percent
Sample	108	72.0%	42	28.0%
Length of delay in seeking advocacy support:		$\bar{x}$ 20.4 (5.4)		$\bar{x}$ 9.1 (3.0)
1–5 years	0	-	5	11.9%
6–10 years	5	4.62%	26	61.9%
11–15 years	17	15.7%	11	26.1%
16–20 years	32	29.6%	0	-
21+ years	54	50.0%	0	-
Length of service:		$\bar{x}$ 8.1 (6.7)		$\bar{x}$ 5.5 (3.4)
1–5 years	51	47.2%	24	57.1%
6–10 years	35	32.4%	15	35.7%
11–15 years	12	11.1%	3	7.1%
16–20 years	0	-	0	-
21+ years	10	9.2%	0	-
Service Branch:				
Army	63	58.3%	27	64.2%
Air Force	33	30.5%	12	28.5%
Navy	12	11.1%	3	7.1%
Highest achieved rank:				
Private	51	47.2%	18	42.8%
Corporal	42	38.8%	22	52.3%
Sergeant	8	7.4%	2	4.7%
Warrant Officer	5	4.6%	-	-
Commissioned	2	1.8%	-	-
Age on advocacy engagement:		$\bar{x}$ 53.9 (11.4)		$\bar{x}$ 45.9 (12.8)
18–32	2	1.8%	7	16.6%
33+	106	98.1%	35	83.3%
Discharge type:				
Voluntary	93	86.1%	22	52.3%
Medical grounds	15	13.8%	20	47.6%
Administrative	-	-	-	-
Injury category:				
Physical and Psychological	68	62.9%	17	40.4%
Physical only	40	37.0%	25	59.5%
Psychological only	-		-	-
Deployment status:				
Not deployed	87	80.5%	38	90.4%
Deployed	21	19.4%	4	9.5%

**Table 2:** Univariable analysis of potential confounders by length of delay, stratified by Sex.

Variables/Confounders	Male (*n* = 108) Length of Delay		Female (*n* = 42) Length of Delay		*p* Value
	$\bar{x}$	s	$\bar{x}$	s	
Overall	20.4	5.4	9.1	3.0	<0.001
Length of service:					
1–5 years	19.7	5.8	8.5	3.0	<0.001
6–10 years	21.1	5.9	9.4	2.8	<0.001
11–15 years	20.5	1.2	12.3	1.5	0.004
16–20 years	-	-	-	-	-
21+ years	21.0	4.1	-	-	-
Service Branch:					
Army	21.6	5.7	8.8	2.8	<0.001
Air Force	19.6	4.2	9.2	3.3	<0.001
Navy	16.4	4.2	11.0	4.0	0.123
Highest achieved rank:					
Private	20.6	5.2	8.8	2.9	<0.001
Corporal	19.7	5.6	9.0	3.0	<0.001
Sergeant	23.3	3.2	13.0	1.4	<0.002
Warrant Officer	23.4	1.8	-	-	-
Commissioned	10.5	0.7	-	-	-
Discharge type:					
Medical	14.8	4.2	8.3	2.7	<0.001
Voluntary	21.3	5.0	9.8	3.1	<0.001
Administrative	-	-	-	-	-
Age on advocacy engagement:					
18–32	9.0	1.4	7.2	2.5	0.299
33+	20.6	5.2	9.4	3.0	<0.001
Injury category:					
Physical and Psychological	20.7	4.4	8.5	2.7	<0.001
Physical only	19.9	6.8	9.4	3.2	<0.001
Psychological only	-	-	-	-	-
Deployment status:					
Not deployed	20.4	5.7	9.0	3.0	<0.001
Deployed	20.4	3.7	9.5	3.6	0.005

## Data Availability

The original data presented in the study are openly available in the Charles Sturt University Research Output at https://doi.org/10.26189/fn2y-5y17.
